# Sherpas share genetic variations with Tibetans for high‐altitude adaptation

**DOI:** 10.1002/mgg3.264

**Published:** 2016-11-23

**Authors:** Sushil Bhandari, Xiaoming Zhang, Chaoying Cui, Yangla, Lan Liu, Ouzhuluobu, Baimakangzhuo, Gonggalanzi, Caijuan Bai, Bianba, Yi Peng, Hui Zhang, Kun Xiang, Hong Shi, Shiming Liu, Gengdeng, Tianyi Wu, Xuebin Qi, Bing Su

**Affiliations:** ^1^ State Key Laboratory of Genetic Resources and EvolutionKunming Institute of ZoologyChinese Academy of SciencesKunming650223China; ^2^ Kunming College of Life ScienceUniversity of Chinese Academy of SciencesBeijing100049China; ^3^ Nepal Academy of Science and TechnologyGPO Box: 3323, KhumaltarLalitpurNepal; ^4^ High Altitude Medical Research CenterSchool of MedicineTibetan UniversityLhasa850000China; ^5^ Institute of Primate Translational MedicineKunming University of Science and TechnologyKunming650500China; ^6^ National Key Laboratory of High Altitude MedicineHigh Altitude Medical Research InstituteXining810012China

**Keywords:** Genetic adaptation, high altitude, hypoxia, Sherpa, Tibetan

## Abstract

**Background:**

Sherpas, a highlander population living in Khumbu region of Nepal, are well known for their superior climbing ability in Himalayas. However, the genetic basis of their adaptation to high‐altitude environments remains elusive.

**Methods:**

We collected DNA samples of 582 Sherpas from Nepal and Tibetan Autonomous Region of China, and we measured their hemoglobin levels and degrees of blood oxygen saturation. We genotyped 29 *EPAS1* SNPs, two *EGLN1* SNPs and the TED polymorphism (3.4 kb deletion) in Sherpas. We also performed genetic association analysis among these sequence variants with phenotypic data.

**Results:**

We found similar allele frequencies on the tested 32 variants of these genes in Sherpas and Tibetans. Sherpa individuals carrying the derived alleles of *EPAS1* (rs113305133, rs116611511 and rs12467821), *EGLN1* (rs186996510 and rs12097901) and TED have lower hemoglobin levels when compared with those wild‐type allele carriers. Most of the *EPAS1* variants showing significant association with hemoglobin levels in Tibetans were replicated in Sherpas.

**Conclusion:**

The shared sequence variants and hemoglobin trait between Sherpas and Tibetans indicate a shared genetic basis for high‐altitude adaptation, consistent with the proposal that Sherpas are in fact a recently derived population from Tibetans and they inherited adaptive variants for high‐altitude adaptation from their Tibetan ancestors.

## Introduction

Sherpas living in Khumbu region of Nepal are renowned for their superior capacity for climbing Himalayas. Most of the Sherpa people are involved in mountaineering field as climbers, porters, and trekking guides and have displayed extraordinary adaptive behavior at high altitude. Such distinctive traits seen in Sherpa staying permanently at high altitude are lower ventilatory response (Lahiri et al. 1967), larger spirometric values (Havryk et al. 2002), relatively lower hemoglobin concentrations (Adams and Shresta 1974; Beall and Reichsman 1984), higher arterial oxygen saturation (Hackett et al. 1980; Keyl et al. 2000), higher affinity of blood for oxygen (Morpurgo et al. 1976), higher heart rate (Pugh and Evans 1962), less psycho‐neurological symptoms (Garrido et al. 1996), and higher work economy (Bastien et al. 2005). These features suggested that Sherpas seem well adapted at Himalayas, therefore an ideal population for studying high‐altitude adaptation (HAA).

There have been extensive genetic studies in Tibetans, and mainly two genes (*EPAS1,* OMIM accession number: 603349 and *EGLN1,*OMIM accession number: 606425) of the HIF pathway were reported to undergone positive selection for high‐altitude adaptation. The *EPAS1* gene encodes HIF‐2*α* and the *EGLN1* gene encodes HIF prolyl 4‐hydroxylase 2 (PHD2). The adaptive mutations within these two genes may regulate the hemoglobin levels in high‐altitude natives as a strategy of adaptation (Beall et al. 2010; Bigham et al. 2010; Ge et al. 2012; Huerta‐Sánchez et al. 2014; Jeong et al. 2014; Lorenzo et al. 2014; Peng et al. 2011; Simonson 2010; Wang et al. 2011; Xiang et al. 2013; Xu et al. 2011; Yi et al. 2010). In addition, a recent study (Lou et al. 2015) identified a novel Tibetan‐enriched deletion (TED), where a 3.4‐kb deletion occurred at 80 kb downstream of *EPAS1* in about 90% of Tibetans, but absent or extremely rare in other world populations including Han Chinese, implying its possible role in HAA. For Sherpas, previous studies have tested a limited number of sequence variants of several candidate genes likely involved in HAA, such as *HIF‐1α* (Suzuki et al. 2003), *eNOS* (Droma et al. 2006), *ACE* (Droma et al. 2008), *EPAS1* (Hanaoka et al. 2012; Jeong et al. 2014), *EGLN1*,* HYOU1*, and *HMBS* (Jeong et al. 2014). Among these different candidate genes, we genotyped *EPAS1*,* EGLN1*, and TED in Sherpas for knowing their genetic cause of superior climbing ability in Himalayas. Previous studies (Bhandari et al. 2015) demonstrated that Sherpas is a recently(<1500 years ago) derived sublineage of Tibetans as reflected by the shared mitochondrial DNA (maternal) and Y chromosome (paternal) lineages between them. Here, we further tested *EPAS1*,* EGLN1*, and TED region in Sherpas to see if they share similar genetic variants on these genes with Tibetans or they have different patterns.

We collected 582 DNA samples from Sherpas staying permanently at highland villages of both Nepal and Tibetan Autonomous Region of China. Additionally, we measured hemoglobin level and degree of oxygen saturation level of 297 Nepalese Sherpas. We genotyped 31 genetic variants of the two key HAA genes (*EPAS1* and *EGLN1*) as well as the 3.4 kb deletion locus (TED) in Sherpas. Our results indicated highly similar adaptive allele frequencies of all tested loci in Sherpas when compared with Tibetans. In contrast, there are sharp allelic divergences among these loci between the Himalayan populations (Sherpas and Tibetans) and lowland populations (Han Chinese, Europeans and Africans), supporting a shared genetic basis for HAA between Sherpas and Tibetans as proposed previously (Foll et al. 2014; Jeong et al. 2014; Jha et al. 2016).

## Material and Methods

### Ethical compliance

The protocols of this study were approved by the Internal Review Boards of Kunming Institute of Zoology, Chinese Academy of Sciences and Nepal Health Research Council, Kathmandu, Nepal. All participants provided written informed consent for this study.

### DNA samples

DNA samples were extracted from blood of 582 unrelated Sherpa individuals staying permanently (>2800 m) in Khumbu region of Nepal and Zhangmu Town (bordering Nepal) of Tibetan Autonomous Region of China. Sherpa ethnicity was confirmed based on their self‐reported parents and grandparents origin. Besides blood sample collection, we also measured hemoglobin and arterial oxygen saturation level of 297 healthy adult Sherpas (126 males and 171 females) residing in highland villages of Khumbu region, Nepal. The arterial oxygen saturation (SaO_2_) was recorded using a hand‐held pulse oximeter (Nellcor NPB‐40, CA) after the individuals take a rest for 5–10 min. A HemoCueHb 201+ analyzer (Angelholm, Sweden) was used to measure hemoglobin of fingertip capillary blood.

### Genotyping of *EPAS1*,* EGLN1*, and TED

Genotyping of selected *EPAS1* (Genbank reference sequence: NC_000002.12) SNPs was done by partial sequencing method covering the respective genomic region of these SNPs. Primers were designed using Primer3 software and genotyping was performed using Sanger sequencing on an ABI 3730 sequencer (Applied Biosystems, Foster City, CA, USA). The LD map of *EPAS1* was constructed using Haploview version 4.1(Barrett et al. 2005). Similarly, genotyping of two missense mutations (rs12097901G and rs186996510C) of *EGLN1* (Genbank reference sequence: NC_000001.11) was done using SNaPshot method on an ABI 3730 sequencer (Applied Biosystems). The SNaPshot method was applied as described previously (Xiang et al. 2013). The 5.5 kb resequencing of *EGLN1* in 50 Sherpa samples was also done using Sanger sequencing on an ABI 3730 sequencer (Applied Biosystems). In addition, genotyping of TED was done following the method described in the previous study (Lou et al. 2015).

### Genetic association analysis

We collected hemoglobin and arterial oxygen saturation level data along with blood samples from 297 healthy adult Sherpas (126 men and 171 women) for genetic association analysis. The three different genotypes of TED were marked as a biallelic marker (zero copy, one copy, and two copies), and the association analysis was done like other SNPs genotype using linear regression with an additive genetic model in PLINK v1.07 (Purcell et al. 2007). We also tested Hardy–Weinberg equilibrium (HWE) for the 14 loci and no deviation was detected.

### Haplotype network analysis

The 28 *EPAS1* SNPs and 17 *EGLN1* SNPs were used to construct haplotype network of Sherpas, Tibetans, and five other populations from the 1000 Human Genomes Projects. The haplotype reconstruction was done using PHASE program embedded in DnaSP Version 5 (Librado and Rozas 2009). The median joining network was constructed using NETWORK 4.6.1.0, Fluxus Engineering (Bandelt et al. 1999).

### *F*_*ST*_ calculation

The unbiased estimates of *F*_*ST*_ was calculated using the method described previously (Weir and Cockerham 1984). We measured the genetic divergence of *EPAS1* SNPs between Sherpas and other populations from the 1000 Genomes Project (CHB, JPT, CEU, and YRI).

## Results

### Sherpas share similar frequencies of the adaptive *EPAS1* variants with Tibetans

*EPAS1* is one of the key HAA genes identified in Tibetans (C. M. Beall et al. 2010; Huerta‐Sánchez et al. 2014; Peng et al. 2011). Previously, we conducted resequencing of the entire 94 kb gene region of *EPAS1* in 50 Tibetans, and we observed many single‐nucleotide polymorphisms (SNPs) showing deep allelic divergence (*F*_*ST*_ >0.45) between Tibetans and Han Chinese (Peng et al. 2011), suggesting that *EPAS1* has undergone strong Darwinian positive selection at high altitude leading to the enrichment of adaptive sequence variants in Tibetans. Among the 82 deeply diverged SNPs (*F*_*ST*_ >0.45), majority of them are located in three major linked blocks of *EPAS1*. Since SNPs lying in the same block are tightly linked (*r* ^2^ >0.8) to each other, we selected one or two SNPs from each block when performing genotyping of *EPAS1* SNPs in Sherpas. In total, 29 out of the 82 SNPs were genotyped in 50 randomly selected Sherpa individuals from Nepal. As shown in Table 1, all 29 SNPs have high frequencies (~49–82%) of the derived alleles (presumably the adaptive alleles), highly similar with the reported allele frequencies in Tibetans (Peng et al. 2011), but deeply diverged from the lowland populations of the 1000 Genomes Project (Abecasis 2012)(CHB: Han Chinese in Beijing, JPT: Japanese in Tokyo, CEU: Utah residents with northern and western European ancestry and YRI: Yoruba in Ibadan, Nigeria).

**Table 1:** Comparison of allele frequencies of 29 *EPAS1* SNPs between highland (Sherpa and Tibetans) and lowland populations (CHB, JPT, CEU, and YRI)

Position Chr 2‐GRCh37	SNP ID	Allele		Frequency of derived allele					
		Ancestral	Derived	Sherpa (50)	TIB (50)	CHB (97)	JPT (89)	CEU (85)	YRI (88)
46550132	rs116088026	A	C	0.51	0.58	0.00	0.00	0.00	0.06
46552202	rs149594770	T	A	0.54	0.51	0.01	0.00	0.00	0.03
46552352	rs140067727	T	C	0.49	0.51	0.01	0.00	0.00	0.02
46553044	rs113305133	A	G	0.51	0.58	0.01	0.00	0.00	0.03
46565091	rs12614710	G	T	0.52	0.42	0.07	0.04	0.44	0.05
46567916	rs115321619	G	A	0.67	0.74	0.01	0.00	0.01	0.19
46568680	rs73926263	A	G	0.64	0.73	0.01	0.00	0.00	0.07
46569017	rs73926264	A	G	0.67	0.73	0.01	0.00	0.00	0.07
46569770	rs73926265	G	A	0.73	0.73	0.01	0.00	0.00	0.08
46570342	rs55981512	G	A	0.75	0.73	0.01	0.00	0.00	0.04
46571017	rs149306391	C	G	0.75	0.73	0.00	0.00	0.00	0.00
46575388	rs4953354	A	G	0.76	0.79	0.12	0.15	0.18	0.19
46576918	rs76242811	T	C	0.75	0.75	0.01	0.00	0.00	0.10
46577251	rs188801636	T	C	0.74	0.75	0.01	0.00	0.00	0.00
46577299	rs6544889	A	G	0.82	0.81	0.14	0.10	0.54	0.18
46577797	SNP155	T	C	0.76	0.75	—	—	—	—
46583581	rs189807021	G	A	0.74	0.75	0.01	0.00	0.00	0.00
46588019	rs150877473	C	G	0.74	0.75	0.01	0.00	0.00	0.00
46588331	rs142826801	G	C	0.74	0.75	0.01	0.00	0.00	0.00
46589032	rs74898705	C	T	0.74	0.75	0.01	0.00	0.03	0.17
46592807	rs61151542	C	T	0.69	0.74	0.01	0.00	0.03	0.19
46594122	rs141366568	A	G	0.69	0.74	0.01	0.00	0.00	0.00
46597756	rs116062164	A	C	0.69	0.76	0.01	0.00	0.04	0.00
46598025	rs141426873	C	G	0.69	0.74	0.01	0.00	0.00	0.00
46600030	rs116611511	A	G	0.7	0.74	0.01	0.00	0.00	0.10
46600661	rs58160876	A	C	0.7	0.74	0.01	0.00	0.00	0.10
46600894	rs12467821	T	C	0.79	0.78	0.15	0.14	0.52	0.10
46609966	rs11690951	A	T	0.68	0.73	0.20	0.22	0.52	0.06
46615955	rs56161503	G	A	0.68	0.73	0.08	0.10	0.49	0.05

**Figure 1:**
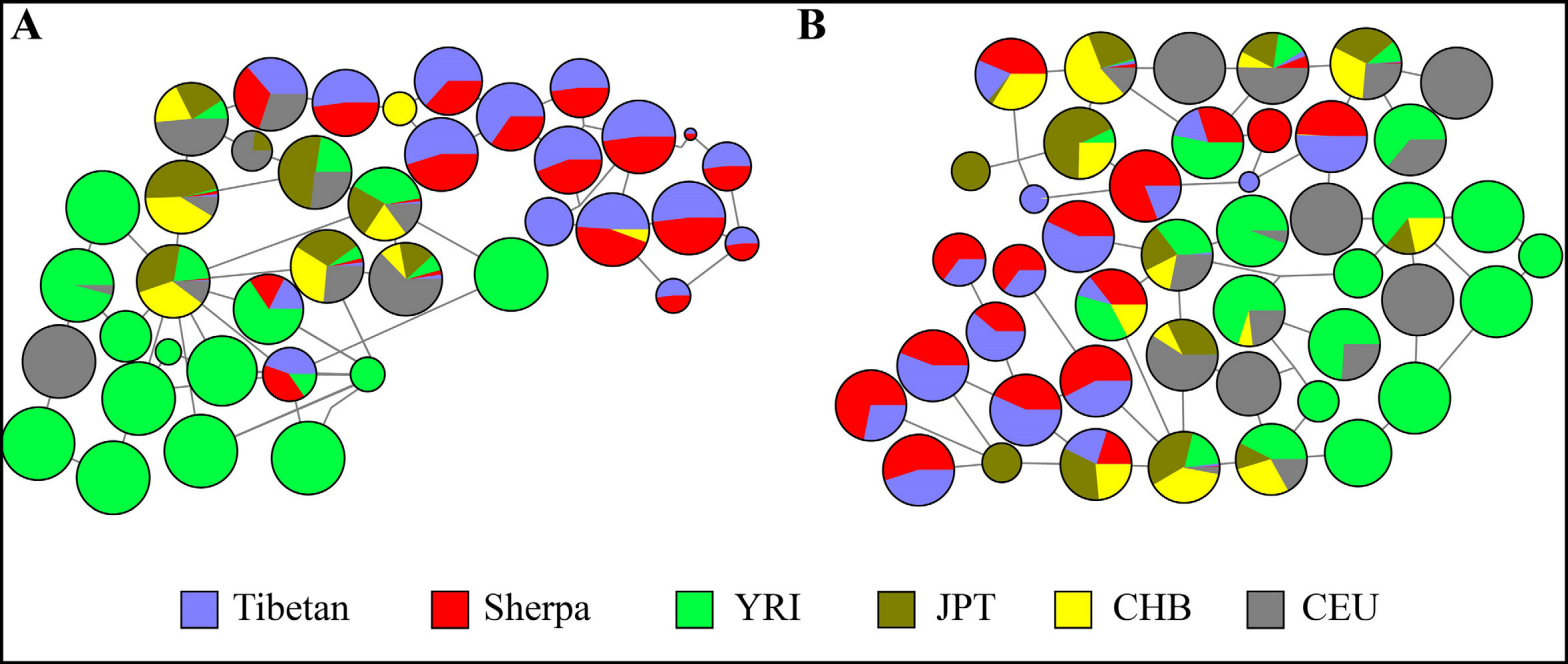
The haplotype networks of *EPAS1* and *EGLN1* among Sherpa and other populations (CHB, CEU, JPT, YRI, and Tibetans). A total of 28 and 17 SNPs were used in network construction for *EPAS1* and *EGLN1*, respectively.

Furthermore, we constructed a haplotype network using these SNPs among Sherpas, Tibetans and other lowland populations (CHB, JPT, CEU, and YRI). It is clearly seen that Sherpas and Tibetans shared almost all *EPAS1* haplotypes, which are extremely rare in Han Chinese, and absent in other world populations (Fig. 1A). Thus, Sherpas and Tibetans might share the same adaptive genetic variants of *EPAS1*, consistent with previous studies (Hanaoka et al. 2012; Jeong et al. 2014).

### TED is highly prevalent in Sherpas

A 3.4‐kb deletion (TED) located 80 kb downstream of *EPAS1* gene is highly prevalent in Tibetans (90%), but rare in Han Chinese and other world populations (3–7%), suggesting its potential role in HAA (Lou et al. 2015). We genotyped the TED locus in 582 Sherpas, and we found that 94% of them are TED carriers with 73% of them being TED homozygotes and 21% TED heterozygotes. The allele frequency of TED in Sherpas (83.5%) is similar with that in Tibetans (90%), consistent with the pattern seen for the *EPAS1* sequence variants.

### Prevalence of *EGLN1* missense variants in Sherpas

Previous studies of *EGLN1* have identified two missense mutations (rs12097901G>C, D4E and rs186996510G>C, S127C) with large allelic divergence between Tibetans and other world populations (Felipe R Lorenzo et al. 2014; Xiang et al. 2013). These two *EGLN1* SNPs were characterized previously (Felipe R Lorenzo et al. 2014) as functional variants for Tibetan high‐altitude adaptation. We genotyped these two SNPs in 582 Sherpas, and it turned out that the adaptive alleles of these two SNPs are also prevalent in Sherpas (67% and 61%, respectively), similar with the reported frequencies in Tibetans (79% and 65–75%, respectively) (Felipe R Lorenzo et al. 2014; Xiang et al. 2013). Similarly, as seen for the *EPAS1* variants, the *EGLN1* adaptive alleles are also rare in Han Chinese and other world populations (0.5–2.3%)(Xiang et al. 2013), again suggesting shared adaptive variants between Sherpas and Tibetans.

We also resequenced a 5.5 kb fragment flanking the two *EGLN1* SNPs in the 50 randomly selected Sherpa samples. A total of 17 SNPs in the 5.5 kb region were used to construct haplotype network, together with data from Tibetans (Xiang et al. 2013) and other world populations (Abecasis 2012)(CHB, JPT, CEU and YRI). The haplotype network analyses clearly indicate that Sherpas and Tibetans share distinct haplotypes of *EGLN1*, which are different from those haplotypes prevalent in other populations (Fig. 1B). Collectively, our data of *EPAS1*, TED, and *EGLN1* all support that there might be shared genetic variants responsible for HAA between Sherpas and Tibetans.

### Genetic association analysis of hemoglobin and blood oxygen saturation in Sherpas

**Figure 2:**
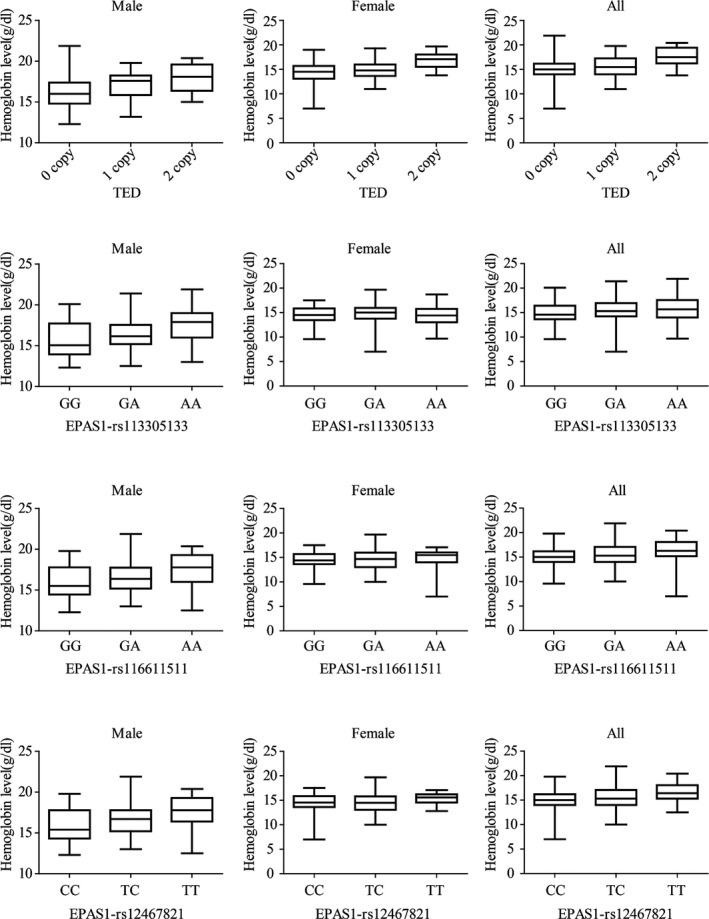
The box plot showing comparison of hemoglobin levels of different genotypes of TED and three *EPAS1* SNPs (rs113305133, rs116611511, and rs12467821) in Sherpas.

To test if the sequence variants contribute to the known adaptive traits in Sherpas, we conducted association studies with hemoglobin concentration and blood oxygen saturation level in 297 Sherpas from Nepal (126 males and 171 females). A total of 14 variants (11 *EPAS1* SNPs, two *EGLN1* SNPs and TED) were genotyped among 297 Nepalese Sherpas for association analysis. As expected, six of the 11 *EPAS1* SNPs showed significant association with hemoglobin concentration (*P* < 0.05, after Bonferroni correction) (Table 2). The significant SNPs reported previously in Tibetans (Beall et al. 2010) and Sherpa (Jeong et al. 2014) were replicated in our present Sherpa studies too. When males and females were analyzed separately, three *EPAS1* SNPs (rs113305133**,** rs116611511, and rs12467821) showed significant association in males but none of these tested *EPAS1* SNPs showed significant association in females (Table 2). Notably, Sherpa individuals carrying the adaptive alleles of the *EPAS1* SNPs tend to have lower hemoglobin levels when compared with those wild‐type allele carriers (Fig. 2), consistent with the result in Tibetans (Beall et al. 2010). Interestingly, two *EPAS1* SNPs (rs116611511 and rs12467821) showing significant association with hemoglobin in Sherpas are tightly linked with the previously proposed 2.5 kb *EPAS1* motif of Denisovan introgression in Tibetans (Huerta‐Sánchez et al. 2014).

**Table 2:** Association analysis of 14 variants (11 *EPAS1* SNPs, 2 *EGLN1* SNPs, and TED) with hemoglobin and blood oxygen saturation in Nepalese Sherpas

Sample	Gene	SNP	Effect allele	Hemoglobin level				Degree of blood oxygen saturation			
				BETA	SE	*P*	Corrected *P*	BETA	SE	*P*	Corrected *P*
Male *N* = 126	*EPAS1*	rs149594770	A	0.629	0.257	0.016	0.204	−0.0022	0.0032	0.490	/
	*EPAS1*	rs140067727	C	−0.671	0.275	0.016	0.214	0.0001	0.0030	0.985	/
	*EPAS1*	**rs113305133**	G	−0.901	0.244	3.30E‐04	**0.004**	−0.0020	0.0030	0.518	/
	*EPAS1*	rs149306391	G	0.617	0.260	0.019	0.252	0.0026	0.0033	0.426	/
	*EPAS1*	rs4953354	G	0.468	0.283	0.101	/	−0.0001	0.0035	0.983	/
	*EPAS1*	rs188801636	C	0.610	0.243	0.013	0.172	0.0007	0.0030	0.819	/
	*EPAS1*	rs6544889	G	0.599	0.239	0.013	0.174	−0.0002	0.0029	0.947	/
	*EPAS1*	SNP155	C	0.196	0.193	0.311	/	−0.0001	0.0023	0.959	/
	*EPAS1*	**rs116611511**	G	0.722	0.233	0.002	**0.032**	0.0003	0.0029	0.929	/
	*EPAS1*	rs58160876	C	0.665	0.239	0.006	0.081	0.0003	0.0029	0.920	/
	*EPAS1*	**rs12467821**	C	0.806	0.252	0.002	**0.023**	−0.0006	0.0031	0.854	/
	TED	**Deletion**	Zero copy	0.930	0.262	0.001	**0.007**	0.0012	0.0033	0.710	/
	*EGLN1*	rs186996510	C	−0.173	0.251	0.491	0.982	0.0015	0.0030	0.617	/
	*EGLN1*	rs12097901	G	−0.154	0.252	0.543	**/**	0.0002	0.0030	0.959	/
Female *N* = 171	*EPAS1*	rs149594770	A	0.023	0.213	0.916	/	−0.0023	0.0022	0.302	/
	*EPAS1*	rs140067727	C	0.083	0.233	0.723	/	0.0040	0.0022	0.072	0.930
	*EPAS1*	rs113305133	G	−0.028	0.217	0.896	/	0.0048	0.0022	0.030	0.388
	*EPAS1*	rs149306391	G	0.652	0.244	0.008	0.106	−0.0015	0.0026	0.565	/
	*EPAS1*	rs4953354	G	−0.128	0.248	0.607	/	−0.0012	0.0026	0.654	/
	*EPAS1*	rs188801636	C	0.457	0.237	0.055	0.718	0.0005	0.0025	0.848	/
	*EPAS1*	rs6544889	G	0.359	0.241	0.138	/	−0.0009	0.0025	0.720	/
	*EPAS1*	SNP155	C	0.246	0.176	0.165	/	−0.0013	0.0018	0.478	/
	*EPAS1*	rs116611511	G	0.286	0.235	0.227	/	0.0005	0.0024	0.835	/
	*EPAS1*	rs58160876	C	0.350	0.234	0.136	/	0.0005	0.0024	0.832	/
	*EPAS1*	rs12467821	C	0.406	0.247	0.103	/	0.0003	0.0026	0.900	/
	TED	**Deletion**	Zero copy	1.004	0.276	3.63E‐04	**0.005**	−0.0010	0.0029	0.727	/
	*EGLN1*	rs186996510	C	0.323	0.192	0.094	0.189	0.0002	0.0021	0.924	/
	*EGLN1*	rs12097901	G	0.333	0.208	0.111	0.222	−0.0010	0.0024	0.656	/
All *N* = 297	*EPAS1*	rs149594770	A	0.293	0.183	0.110	/	−0.0023	0.0018	0.207	/
	*EPAS1*	rs140067727	C	−0.271	0.197	0.170	/	0.0024	0.0018	0.198	/
	*EPAS1*	rs113305133	G	−0.430	0.182	0.019	0.242	0.0020	0.0018	0.275	/
	*EPAS1*	**rs149306391**	G	0.722	0.198	3.16E‐04	**0.004**	0.0002	0.0020	0.932	/
	*EPAS1*	rs4953354	G	0.211	0.208	0.312	/	−0.0009	0.0021	0.654	/
	*EPAS1*	**rs188801636**	C	0.674	0.185	3.25E‐04	**0.004**	0.0001	0.0019	0.956	/
	*EPAS1*	**rs6544889**	G	0.630	0.185	0.001	**0.010**	−0.0010	0.0019	0.589	/
	*EPAS1*	SNP155	C	0.338	0.142	0.018	0.235	−0.0011	0.0014	0.443	/
	*EPAS1*	**rs116611511**	G	0.643	0.182	4.66E‐04	**0.006**	−0.0001	0.0019	0.966	/
	*EPAS1*	**rs58160876**	C	0.640	0.183	0.001	**0.007**	0.0000	0.0019	0.982	/
	*EPAS1*	**rs12467821**	C	0.750	0.194	1.31E‐04	**0.002**	−0.0006	0.0020	0.761	/
	TED	**Deletion**	Zero copy	1.032	0.209	1.32E‐06	**1.71E‐05**	0.0000	0.0022	0.983	/
	*EGLN1*	rs186996510	C	0.051	0.166	0.759	/	0.0008	0.0018	0.629	/
	*EGLN1*	rs12097901	G	0.029	0.174	0.866	/	−0.0003	0.0019	0.872	/

Bold values indicated those p values <0.05.

Furthermore, TED showed significant association with hemoglobin in both males and females, and it stands the most significant variant in the combined sample (*P* = 1.71 × 10^−5^, after Bonferroni correction) (Table 2). Similar with the *EPAS1* SNPs, the TED carriers have lower hemoglobin levels than the nondeletion carriers (Fig. 2). Taken together, these data suggest that *EPAS1* and TED might have contributed to hemoglobin regulation in Sherpas by keeping a relatively low hemoglobin concentration at high altitude. We did not observe significant association of the tested variants (Table 2) with blood oxygen saturation, implying that there might be different genetic mechanisms of regulating hemoglobin and blood oxygen saturation.

## Discussion

*EPAS1* and *EGLN1* are two top candidate genes reported in previous studies for HAA in Tibetans (Beall et al. 2010; Lorenzo 2010; Lorenzo et al. 2014; Peng et al. 2011; Scheinfeldt and Tishkoff 2013; Simonson 2010; Xiang et al. 2013; Xu et al. 2011; Yi et al. 2010). Additionally, a recent study (Lou et al. 2015) identified TED with high frequency in Tibetans, but rare or absent in lowland populations though whether TED is functionally related with *EPAS1* is unknown because it is located 80 kb downstream of *EPAS1*. We previously showed that Sherpas are likely a recently derived population from Tibetans (<1500 years ago) based on their shared mtDNA and Y chromosome lineages (Bhandari et al. 2015). Hence, we propose that they might share similar genes/mutations for HAA, which was confirmed by our analysis of 32 variants of the top candidate HAA polymorphisms (*EPAS1*, TED, and *EGLN1*). Our comparative studies between these two highland populations (Sherpas and Tibetans) demonstrated sharing of similar genetic variants in *EPAS1*,* EGLN1*, and TED, a further support for the biological significance of these HAA genes for future functional studies.

The observed significant association of *EPAS1* SNPs and TED with hemoglobin levels suggests the functional importance of *EPAS1* for high‐altitude adaptation because a relatively low hemoglobin level can prevent onset of polycythemia that impairs tissue blood flow and oxygen delivery (Beall 2007). Our association studies did not find any significant *EPAS1* SNPs in females when analyzed separately in males and females. It might be due to either the differences in gender‐specific variants responsible for maintaining hemoglobin concentration in males and females or our limited phenotypic data on pregnancy, menstruation, and breast feeding for association studies which may affects hemoglobin levels in females.

The 2.5 kb *EPAS1* motif initially identified in Tibetans was proposed to emerge as the result of Denisovan introgression, potentially responsible for high‐altitude adaptation (Huerta‐Sánchez et al. 2014). But it is still difficult to pinpoint the causal mutation(s) since multiple SNPs in *EPAS1* have large allelic divergence between Tibetans/Sherpas and lowland populations (e.g., Han Chinese), and show significant association with hemoglobin levels. Furthermore, in Sherpas the deletion polymorphism (TED), 80 kb downstream of *EPAS1* showed a stronger signal of association with hemoglobin than the *EPAS1* SNPs. But TED is not caused by Denisovan introgression since it is absent in the Denisovan genome (Lou et al. 2015). Hence, whether Tibetans and Sherpas acquired their adaptive ability of living in Himalayas through Denisovan introgression need to be tested in future studies.

In conclusion, we observed shared adaptive variants of the key HAA genes (*EPAS1*,* EGLN1* and TED) between Sherpas and Tibetans. The superior capacity of Sherpas for mountain climbing as a reflection of adaptation to high altitude was inherited from their Tibetan ancestors who have been living in the Himalayas for a long time.

## Competing Financial Interests

The authors declare no competing financial interests.

## Web Resources

1000 Genomes Projects, http://www.1000genomes.org/; UCSC Genome Browser, http://genome.ucsc.edu/; Primer3, http://frodo.wi.mit.edu/cgi-bin/primer3/primer3_www.cgi.
